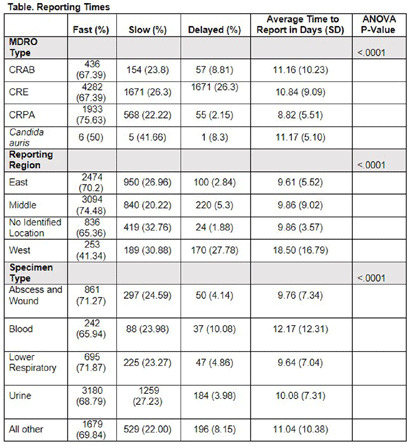# Variability of MDRO Reporting Across Tennessee Microbiology Laboratories

**DOI:** 10.1017/ash.2024.332

**Published:** 2024-09-16

**Authors:** Matthew Lokant, Christopher Wilson, Tom Talbot, Priscilla Pineda, Erin Hitchingham, Melphine Harriott, Raquel Villegas, Kaleb Wolfe, Milner Owens Staub

**Affiliations:** Vanderbilt University Medical Center; Tennessee Department of Health; Vanderbilt University School of Medicine; TN Department of Health HAI/AR Program; state of TN

## Abstract

**Background:** Identification and timely reporting of multi-drug resistant organisms (MDROs) drives efficacy of infection prevention efforts. Data on MDRO reporting timeliness and inter-facility variability are limited. Facility-dependent variability in MDRO reporting across Tennessee was examined to identify opportunities for MDRO surveillance improvement. **Methods:** Data for reported Tennessee MDROs including carbapenem-resistant Enterobacterales (CRE), carbapenem-resistant Acinetobacter baumannii (CRAB), Carbapenem-resistant Pseudomonas aeruginosa (CRPA) and Candida auris, were obtained from the southeast regional Antibiotic Resistance Laboratory Network (ARLN) from 2018-2022, excluding screening and colonization specimens. Variance in days accrued from specimen collection to ARLN receipt was analyzed using one-way analysis of variance (ANOVA) with Tukey’s test (SAS 9.4). Facilities were categorized as fast (1-10 days), slow (11-20 days), or delayed (21-100 days) reporters. **Results:** There were 9,569 MDRO isolates reported. CRPA was reported faster than other MDROs (p < 0.001), while specimens from West Tennessee compared to other regions (p < 0.001) (Figure) and blood cultures compared to other specimens were reported more slowly (p < 0.001) (Table). There was no difference in reporting times for facilities using on-site microbiology laboratories versus reference laboratories (P = 0.062). **Conclusion:** MDRO reporting times varied across Tennessee by region, specimen, and organism. Future work to elucidate drivers of variability will consist of surveys and focused interviews with laboratory personnel to identify shared and unique barriers and opportunities for improvement.

**Figure:**
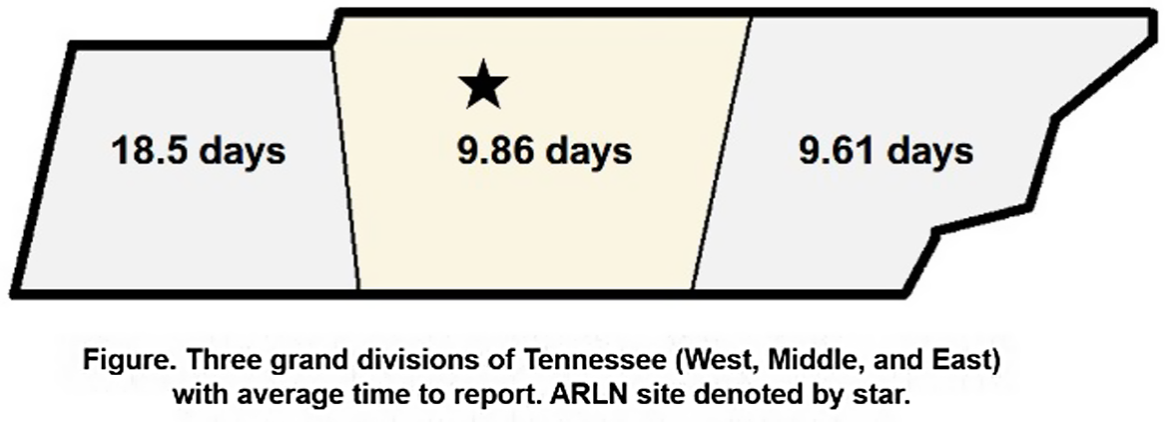

**Figure:**